# Comparative transcriptome and metabolome provides new insights into the regulatory mechanisms of accelerated senescence in litchi fruit after cold storage

**DOI:** 10.1038/srep19356

**Published:** 2016-01-14

**Authors:** Ze Yun, Hongxia Qu, Hui Wang, Feng Zhu, Zhengke Zhang, Xuewu Duan, Bao Yang, Yunjiang Cheng, Yueming Jiang

**Affiliations:** 1 Key Laboratory of Plant Resources Conservation and Sustainable Utilization, South China Botanical Garden, Chinese Academy of Sciences, Guangzhou 510650, P.R. China; 2 Key Laboratory of Horticultural Plant Biology, Ministry of Education, Huazhong Agricultural University, Wuhan 430070, P.R. China

## Abstract

Litchi is a non-climacteric subtropical fruit of high commercial value. The shelf life of litchi fruit under ambient conditions (AC) is approximately 4–6 days. Post-harvest cold storage prolongs the life of litchi fruit for up to 30 days with few changes in pericarp browning and total soluble solids. However, the shelf life of litchi fruits at ambient temperatures after pre-cold storage (PCS) is only 1–2 days. To better understand the mechanisms involved in the rapid fruit senescence induced by pre-cold storage, a transcriptome of litchi pericarp was constructed to assemble the reference genes, followed by comparative transcriptomic and metabolomic analyses. Results suggested that the senescence of harvested litchi fruit was likely to be an oxidative process initiated by ABA, including oxidation of lipids, polyphenols and anthocyanins. After cold storage, PCS fruit exhibited energy deficiency, and respiratory burst was elicited through aerobic and anaerobic respiration, which was regulated specifically by an up-regulated calcium signal, G-protein-coupled receptor signalling pathway and small GTPase-mediated signal transduction. The respiratory burst was largely associated with increased production of reactive oxygen species, up-regulated peroxidase activity and initiation of the lipoxygenase pathway, which were closely related to the accelerated senescence of PCS fruit.

Litchi is an important economic crop in subtropical China. Because of its high nutritional value, sweet taste and attractive red colour, litchi fruit is strongly favoured and sought by consumers. However, the short shelf life of the litchi fruit under ambient conditions has greatly restricted the development of the litchi industry. Under ambient conditions, litchi fruit are highly perishable, which is characterised by pericarp browning and loss of flavour^1^,^2^. To date, cold storage has proven to be the most efficient method of prolonging the life of litchi fruits^3^ and is widely used in the litchi industry. Although cold storage is effective at increasing the shelf life of litchi fruit for up to approximately 30 days, the subsequent shelf life of the fruit under ambient conditions is very short (<48 h) compared with fruit that had been stored only under ambient conditions^4^.

Fruit ripening and senescence are complex biological processes that are unique to plants. Fruit can be divided into two groups according to their ripening and senescence processes. Climacteric fruit are characterized by ripening-associated increases in respiration and ethylene production rates, and the phytohormone ethylene can act as a major trigger and coordinator of the ripening process. In contrast, no significant increase in phytohormones is observed in non-climacteric fruits^1^. In these fruits, the best fruit quality and flavour are achieved at harvest maturity, followed by a gradual slowing of the fruit senescence process. The little experimental evidence available reveals that factor-associated signalling pathways are the main drivers of the fruit ripening process in non-climacteric fruits. The identification of the regulatory or structural genes controlling fruit ripening and senescence is necessary for the improvement of fruit quality.

Over the past few years, the abscisic acid (ABA) signalling pathway has been investigated extensively^5^. For example, as an intracellular messenger, ABA performs a critical function in improving plant tolerance to cold stress^6^ and activating senescence^7^,^8^. In addition, other signalling molecules are thought to be involved in plant senescence, including ethylene, auxin, reactive oxygen species (ROS), jasmonic acid and salicylic acid^7^,^9^. However, further investigation is needed to determine which of these pathways is plays a central role in the induction of litchi fruit senescence.

ROS play an important role in regulating physiological activities such as plant senescence. Because plant cells are in a state of oxidative stress during senescence, enhanced ROS production can pose a threat by causing lipid peroxidation, protein oxidation, nucleic acid damage, enzyme inhibition and activation of the programmed cell death pathway, which ultimately leads to cell death^10^. ROS accumulation is largely determined by the balance between ROS production and scavenging capacities of the fruit. Shifts in this balance have an impact on the senescence, quality deterioration and loss of marketability of horticultural products^11^. ROS levels are usually controlled by ROS-scavenging antioxidant enzymes, such as superoxide dismutases, catalases and peroxidases^12^. In addition to these enzyme systems, fruit also contains a variety of antioxidants, including phenolic compounds and anthocyanins^13^,^14^.

Pericarp browning is a sensory characteristic of litchi fruit. Fruit with more than 25% pericarp browning is not usually considered to be acceptable by consumers. Pericarp browning is mainly attributable to the degradation of anthocyanins and the oxidation of phenolic compounds by polyphenol oxidase^2^,^14^. Moreover, oxidation of polyphenols by peroxidase in the presence of hydrogen peroxide, as another oxidation pathway, not only removes ROS but also leads to pericarp browning of litchi fruit^15^. Inhibition of lipid peroxidation, which reduces membrane fluidity and increases membrane permeability^15^, delays pericarp browning and extends the storage life of litchi fruit^1^. Most research in the past two decades has focused on the development of litchi preservation technology. However, little attention has been focused on the understanding of the regulatory mechanisms of post-harvest senescence in litchi fruit.

In this study, the transcriptome of the litchi pericarp was assembled; technologies for comparing the transcriptome and metabolome were then applied to investigate the global changes that occur during litchi fruit senescence to provide new insights into its mechanism.

## Results

The shelf life of AC fruit was 4–6 days. Cold temperatures prolonged the storage time of litchi fruit. In this study, litchi fruit that were stored for 14 days at 4 °C and 75–85% relative humidity (RH) exhibited little pericarp browning, but the shelf life of the fruit after removal from cold storage (PCS fruit) was only approximately 1–2 days at 20–25 °C and 75–85% RH. Therefore, the senescence of litchi fruit, as indicated by its shelf life, was significantly accelerated after pre-cold storage compared with normal senescence at ambient temperature. The main aim of the present study was to determine the possible mechanism of accelerated senescence in PCS fruit. A comparative analysis between the transcriptomes and metabolomes of the PCS fruit at 24 h with those at 0 h, named Group 1, was completed; a similar comparative analysis of AC fruits at 4 days with those at 0 days was performed and named Group 2. The up-regulated genes and metabolites in both Groups 1 and 2 were closely associated with litchi fruit senescence as a whole, whereas genes and metabolites that were up-regulated in Group 1 but not Group 2 were mainly associated with the accelerated senescence observed in the PCS fruit.

### Characteristics of litchi fruit senescence under different storage conditions

**Figure 1:**
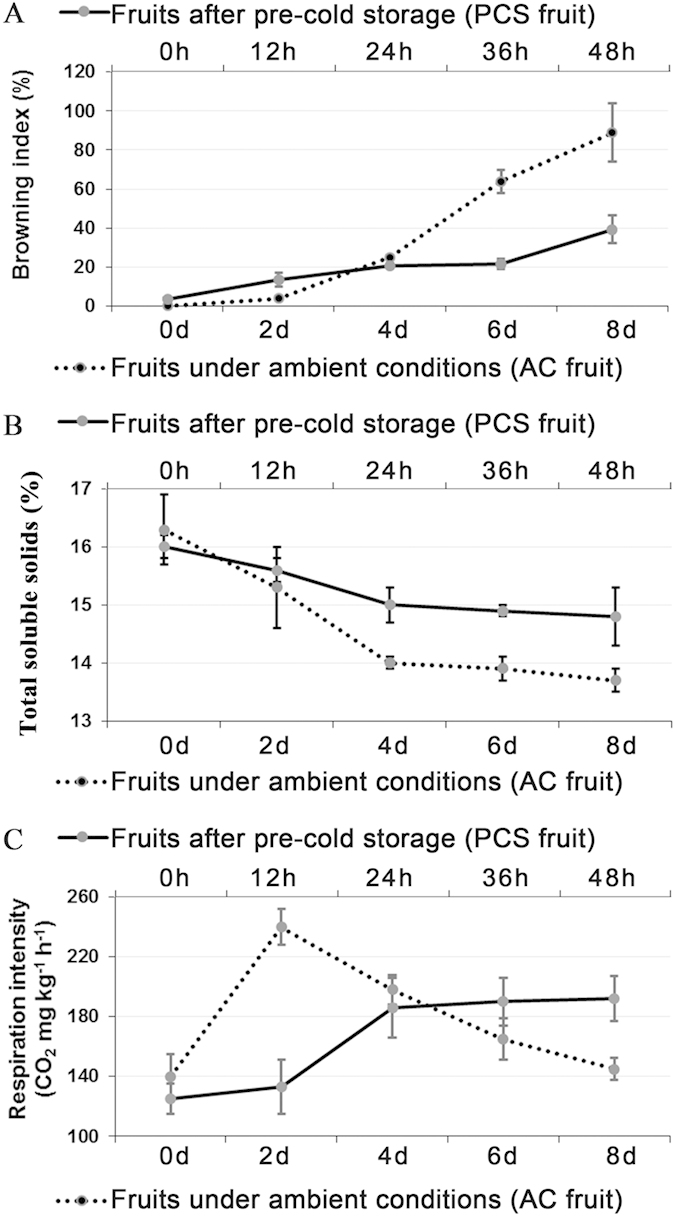
Browning index (**A**), TSS (**B**) and respiration intensity (**C**) during litchi fruit senescence under ambient conditions and shelf-life after pre-cold storage. AC fruits were stored under ambient temperature conditions (approximately 20–25 °C and 75–85% relative humidity) immediately after harvest. After 14 days of cold storage (4 °C and 75–85% relative humidity), PCS fruits were stored under the same ambient temperature conditions mentioned above. The standard errors are presented using error bars.

To identify any differences in senescence between the AC and PCS fruits, the browning index, total soluble solids (TSS) and respiration rate were determined. Browning indices of 20 and 80% were observed in AC fruits after 4 and 8 days of storage, respectively (Fig. 1A). In contrast, cold storage significantly inhibited pericarp browning, indicated by a browning index of 3.57% after 14 days of cold storage (Fig. 1A). However, the fruit placed at ambient temperature after removal from cold storage browned more rapidly (Supplementary Fig. S1). The browning indices of the PCS fruit after 24 and 48 h at ambient temperature following 14 days of cold storage were 20.83 and 39%, respectively (Fig. 1A).

There were no significant differences in the TSS contents of the AC fruit at harvest and the PCS fruit after 14 days of cold storage (Fig. 1B). Thereafter, the TSS content decreased gradually in both the AC and PCS fruit during storage. Interestingly, the rates of decrease in TSS content slowed down in both AC and PCS fruits after 4 days and 24 h, respectively (Fig. 1B).

The respiration rate peaked in the AC fruit after 2 days and then decreased gradually (Fig. 1C). In contrast, the respiration rate in the PCS fruit increased gradually to a plateau by the end of the shelf period (Fig. 1C).

Taken together, the results showed that cold storage effectively slowed down litchi pericarp browning and slowed the decrease in the TSS content. A similar extent of browning was observed in the AC fruit after 4 days and in the PCS fruit after 24 h. The findings revealed a critical comparative point in terms of senescence in 4-day AC fruit and 24-h PCS fruit, which were sampled in subsequent transcriptomic analyses.

### Litchi pericarp transcriptome

Because there is a lack of genomic information about litchi, the transcriptome was sequenced for reference in future comparative analyses. To characterise the transcriptome of the litchi pericarp during senescence, total RNA samples were extracted from the AC fruit after 0 and 4 days of storage at ambient temperature and from the PCS fruit after 0, 24 and 48 hours on the shelf at ambient temperature (after pre-cold storage). After cleaning and quality checks, Illumina paired-end sequencing was run, and 243,174,996 reads were generated, each read averaging 100 or 40 bp in length. Supplementary Table S1 provides a summary of the sequence data. After assembly, 58,638 unigenes of the following lengths were identified: 200−500 bp (61.49%), 500−1000 bp (16.31%), 1000−3000 bp (17.97%) and >3000 bp (4.23%). The assembled sequences were deposited in the NCBI database. After ESTScan analysis, 32,252 coding regions and proteins were identified. A total of 28,013 unigenes were annotated in the nonredundant (NR) database: 16,310 unigenes, 8855 unigenes and 2739 unigenes were annotated in the Clusters of Orthologous Groups (COG), Gene Ontology (GO) and Kyoto Encyclopedia of Genes and Genomes (KEGG) databases, respectively.

### Pericarp transcriptomes of litchi fruit stored under different conditions

**Figure 2:**
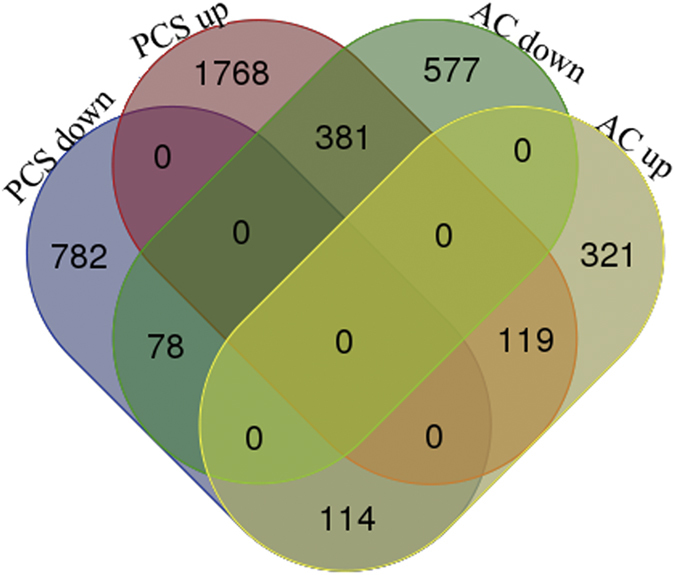
DEGs in senescing litchi fruit under ambient conditions and shelf-life after pre-cold storage. A Venn diagram was constructed. PCS down: genes down-regulated in PCS fruits; PCS up: genes up-regulated in PCS fruits; AC down: genes down-regulated in AC fruits; AC up: genes up-regulated in AC fruits.

Litchi fruit are highly perishable, with a shelf life of approximately 6 days under ambient conditions. Cold temperatures effectively extend the storage life of litchi fruit, but the subsequent shelf life at ambient temperature is only 2 days (Fig. 1). In the present study, the pericarp transcriptomes of fruit stored at ambient temperature and fruit stored at ambient temperature after cold storage were analysed. To increase the reproducibility of the sequencing data, one cDNA library was paired-end sequenced (100 bp), and the other was single-read sequenced (40 bp). An analysis of the statistical significance of the data produced by the two sequencing approaches was performed respectively. The genes that were expressed at significant levels (in both sets of sequencing data) and showed more than a 2-fold change in expression level were used for further analysis. It was determined that a total of 4140 genes were differentially expressed in litchi fruit during senescence. Among them, 1036 genes were down-regulated, and 554 genes were up-regulated in AC fruits at 4 days compared with those at 0 days. In contrast, 2268 genes were up-regulated and 974 genes were down-regulated in PCS fruits at 24 h, compared with those at 0 h. Of the 692 genes that were differentially expressed in both AC and PCS fruit, 119 genes were up-regulated in both AC and PCS fruits, whereas 78 genes were down-regulated in both AC and PCS fruit (Fig. 2). Interestingly, 2149 genes were up-regulated in only the PCS fruit, and 114 genes were specifically down-regulated in only the PCS fruit (Fig. 2).

**Figure 3:**
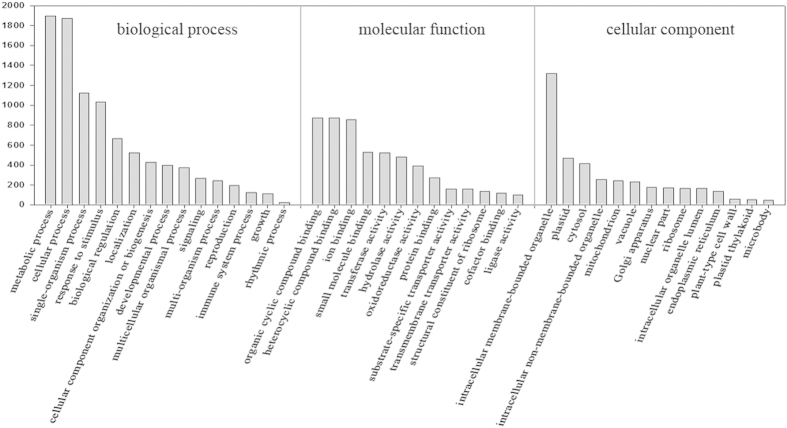
Cluster analysis of DEGs according to GO analysis. GO term assignments were based on significant hits against the NR database for plant species. A total of 4140 differentially expressed genes were clustered using blast2GO, and the analysis was performed with respect to biological processes, molecular functions and cellular components. Level-three data are shown in this figure.

To obtain more information from the large quantities of data, gene function clustering analysis was applied to handle information extraction. Functional analysis of the 4140 differentially expressed genes by Blast2GO was performed across the broad groupings of biological processes, molecular function and cellular components. In biological processes, the genes were distributed among 15 categories (Fig. 3): the largest one being “metabolic process” (1897 genes), followed by “cellular process” (1870 genes), “single-organism process” (1125 genes) and “response to stimulus” (1032 genes). In the molecular function group, the largest category was “organic cyclic compound binding” (872 genes) followed by “heterocyclic compound binding” (872 genes). Interestingly, a large number of these genes were classified as “transferase activity” genes (522), “hydrolase activity” genes (479) and “oxidoreductase activity” genes (391). In the cellular component group, most of the gene functions were clustered into the following categories: “intracellular membrane-bounded organelle” (1319 genes), “plastid” (469 genes), “cytosol” (415 genes), “intracellular non-membrane-bounded organelle” (256 genes), “mitochondrion” (241 genes), “vacuole” (231 genes), “Golgi apparatus” (175 genes), “nuclear part” (170 genes), “ribosome” (167 genes), “intracellular organelle lumen” (165 genes) and “endoplasmic reticulum” (138 genes).

**Figure 4:**
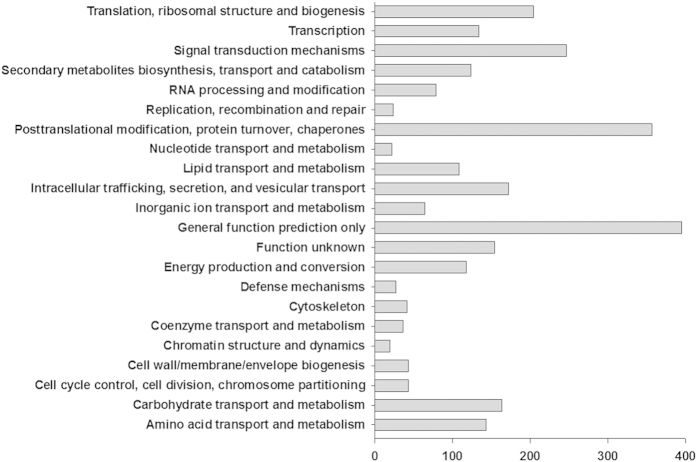
Cluster analysis of DEGs according to KEGG pathways. After comparative transcriptomic analysis, 4140 genes were found to be differentially expressed during litchi fruit senescence. All those genes were classified according to the KEGG pathway database. Clusters of interest were selected for subsequent analysis.

KEGG pathway analysis classified the 4140 differentially expressed genes into 22 categories, of which the largest one was ‘general function prediction only’ (395 genes), followed by ‘post-translational modification, protein turnover and chaperones’ (357 genes), and ‘signal transduction mechanisms’ (247 genes). These results suggest that these genes have pivotal roles in signal transduction and post-translational modification during fruit senescence. The remaining differentially expressed genes were placed in the following categories: ‘carbohydrate transport and metabolism’ (164 genes), ‘amino acid transport and metabolism’ (143 genes), ‘energy production and conversion’ (118 genes), ‘lipid transport and metabolism’ (109 genes) and ‘secondary metabolite biosynthesis, transport and catabolism’ (124 genes) (Fig. 4).

### Comparison of the pericarp metabolome of litchi fruit under different storage conditions

**Figure 5:**
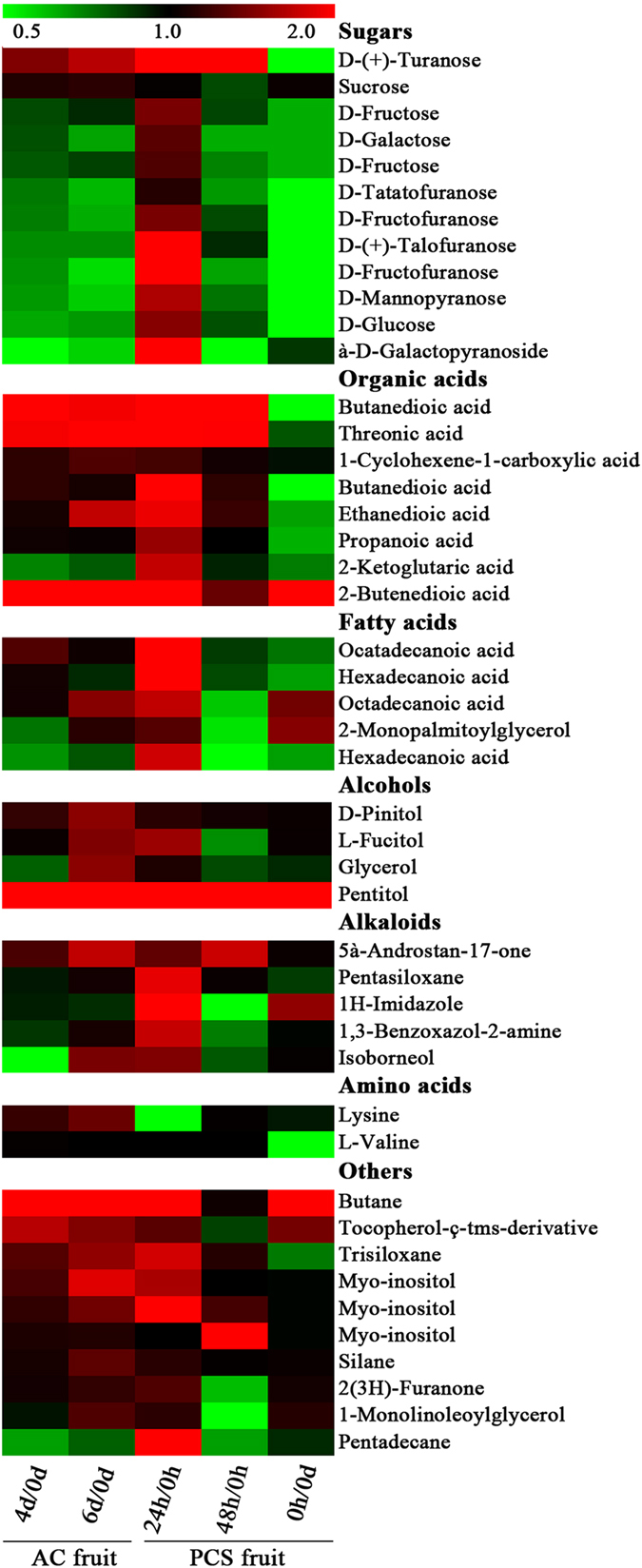
Differentially accumulated primary metabolites in senescing litchi fruits under ambient conditions and shelf life after pre-cold storage. PCS fruit samples (0, 24 and 48 h) and AC fruit samples (0, 4 and 6 days) were used for the differential primary metabolic profiling analysis. Metabolites were detected by GC-MS using ribitol as the internal standard. The data are presented as ratios of the two samples. Up-regulated metabolites are presented in red and down-regulated metabolites in green.

Comparative primary metabolomic analysis was conducted by GC-MS. A total of 65 metabolites were identified using the external standard method and were searched against the GC-MS metabolite database. Among them, 46 metabolites were differentially accumulated during fruit senescence. They were classified into the following seven clusters: sugars (12 metabolites), organic acids (eight metabolites), fatty acids (five metabolites), alcohols (four metabolites), alkaloids (five metabolites), amino acids (two metabolites) and others (ten metabolites). Interestingly, the amounts of most of these metabolites were lower in the PCS fruit at 0 h than in the AC fruit at 0 days (Fig. 5). In contrast, almost all metabolites accumulated in the PCS fruit by 24 h compared with those at 0 h, including sugars, organic acids, fatty acids, alcohols, alkaloids, amino acids and others (Fig. 5). It seems that the primary metabolites of litchi fruit decreased during cold storage but increased sharply after transfer to ambient temperature. Specifically, several metabolites increased significantly in both AC and PCS fruit during senescence, including D-(+)-turanose, butanedioic acid, threonic acid, 5α-androstan-17-one, butane, trisiloxane and myo-inositol (Fig. 5). Apparent differences between the metabolomes of the PCS and AC fruits were observed. For example, the content of octadecanoic acid increased significantly in the 6-day AC fruit compared with those at 0 days but decreased significantly in the 48-h PCS fruit compared with those at 0 h. Similar trends were also observed with *L*-fucitol, glycerol, isoborneol, 2(3H)-furanone and 1-monolinoleoylglycerol (Fig. 5).

### Signal transduction-related genes that were differentially expressed during litchi fruit senescence

**Figure 6:**
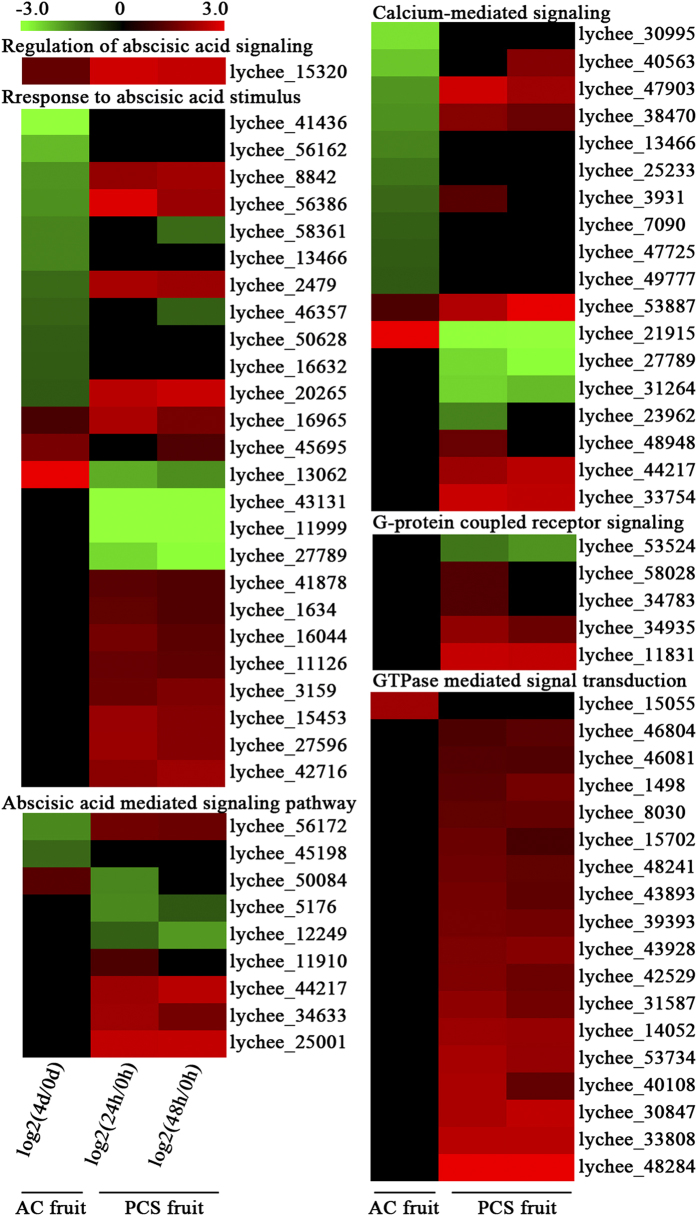
DEGs related to signal transduction in senescing litchi fruit under ambient conditions and shelf-life after pre-cold storage. After cluster analysis, genes related to ABA, calcium ion, G protein and GTPase signal transduction were selected, and the logarithm with base 2 of the ratio between the two samples was calculated. Up-regulated genes are presented in red and down-regulated genes in green.

The 247 signal transduction genes were analysed for functions using BiNGO 2.44. The results suggest that litchi fruit senescence was regulated co-ordinately by various factors, including ABA, *G* protein-coupled receptor proteins, small GTPases and calcium ions (Supplementary Fig. S2). Interestingly, lychee_15320, a gene involved in the ‘regulation of the ABA-mediated signalling pathway’, was up-regulated in both the AC and PCS fruit. However, the trends observed in the ABA-related gene expression profiles varied between the AC and PCS fruit. For example, the genes related to the ABA-mediated signalling pathway and the response to ABA stimulation were up-regulated in the PCS fruit but down-regulated or not significantly altered in the AC fruit (Fig. 6). Thus, the results suggest that senescence was regulated by ABA through the activation of various sets of target genes in the AC and PCS fruits. The only GTPase up-regulated in the AC fruit was lychee_15055. In contrast, 18 small GTPase signalling-related genes were up-regulated in the PCS fruit (Fig. 6). Furthermore, 4 *G protein*-coupled receptor signalling pathway-related genes were up-regulated in the PCS fruit but not in the AC fruit (Fig. 6), suggesting that GTPase and *G* protein might be involved in PCS fruit but not AC fruit senescence. Two calcium ion-signalling genes were up-regulated, and ten genes were down-regulated during AC fruit senescence (Fig. 6); seven genes were up-regulated, and four were down-regulated during PCS fruit senescence (Fig. 6). In short, ABA and calcium ion signalling might be involved in the senescence of both AC and PCS fruits, but small GTPases and *G* proteins were induced only during PCS fruit senescence.

### Genes related to secondary metabolism that were differentially expressed during litchi fruit senescence

Of the genes involved in secondary metabolism, 124 genes were differentially expressed during litchi fruit senescence. Among these genes, 40 genes were down-regulated and 32 genes were up-regulated during AC fruit senescence, whereas 14 genes were down-regulated and 65 genes were up-regulated during PCS fruit senescence. The genes that were up-regulated during AC and PCS fruit senescence were involved in fatty acid oxidation, ABA metabolism, gibberellin metabolism, oxidation and reduction, and oxygen and ROS metabolism (Supplementary Fig. S3). In contrast, many of the genes that were up-regulated during PCS fruit senescence were down-regulated or showed no significant change during AC fruit senescence. These genes were involved in phenylpropanoid, coumarin, lignin and flavonoid metabolism and included lychee_5244, lychee_48526, lychee_28548, lychee_43311, lychee_47963, lychee_21016, lychee_4641, lychee_48170, lychee_14457 and lychee_22413. This result indicated that more dramatic changes occurred in secondary metabolism during PCS fruit senescence than in the AC fruit. Interestingly, some genes related to cytochrome P450 were up-regulated during both AC and PCS fruit senescence or were specifically up-regulated during senescence in the PCS fruit. This finding suggested that cytochrome P450 might play a pivotal role during litchi fruit senescence.

### Lipid transport- and metabolism-related genes that were differentially expressed during litchi fruit senescence

Seven genes involved in lipid transport and metabolism were up-regulated during AC fruit senescence, whereas 72 genes were up-regulated in senescent PCS fruit. Most of these genes were involved in fatty acid oxidation or glycerophospholipid, sterol and isopentenyl metabolism (Supplementary Fig. S4). Several processes of fatty acid oxidation were represented by genes that were up-regulated in the PCS fruit, including α-oxidation, β-oxidation, unsaturated fatty acid β-oxidation and other oxidations. The related genes included lychee_16708, lychee_3591, lychee_4924, lychee_52524, lychee_46445, lychee_29914 and lychee_56255. Interestingly, two genes were detected and predicted to be lipoxygenase, a critical enzyme in lipid peroxidation. Lychee_54651 was up-regulated during PCS fruit senescence, but no significant change in its expression was observed in the AC fruit; lychee_45350 and lychee_52428 were down-regulated during AC fruit senescence, but their expression levels were not significantly changed in the PCS fruit.

### Genes related to oxidation-reduction processes that were differentially expressed during litchi fruit senescence

Genes involved in oxidation-reduction processes accounted for 126 of the genes that were differentially expressed during litchi fruit senescence. A large number of the up-regulated genes were predicted to be peroxidases, most of which were up-regulated specifically in the PCS fruit. Interestingly, there was no significant expression of polyphenol oxidase-related genes during litchi fruit senescence. In addition, two genes that encode a key subunit of nicotinamide-adenine dinucleotide phosphate (NADPH) oxidase, lychee_2233 and lychee_20470, were predicted to be respiratory burst oxidases. In this context, lychee_2233 was up-regulated in the AC and PCS fruits during senescence, whereas lychee_20470 was up-regulated in the PCS fruit and down-regulated in the AC fruit.

### Genes related to protein phosphorylation that were differentially expressed during litchi fruit senescence

Sixteen genes classified as protein phosphorylation genes were down-regulated and four genes were up-regulated during AC fruit senescence. In contrast, 47 protein phosphorylation genes were up-regulated during PCS fruit senescence, including seven calcium signal-related genes, ten serine/threonine protein kinase-related genes, 11 other protein kinase-related genes and two ATP-binding proteins. Thus, it appears that more proteins were phosphorylated in the PCS fruit than in the AC fruit.

### Genes related to energy production and conversion processes that were differentially expressed during litchi fruit senescence

**Figure 7:**
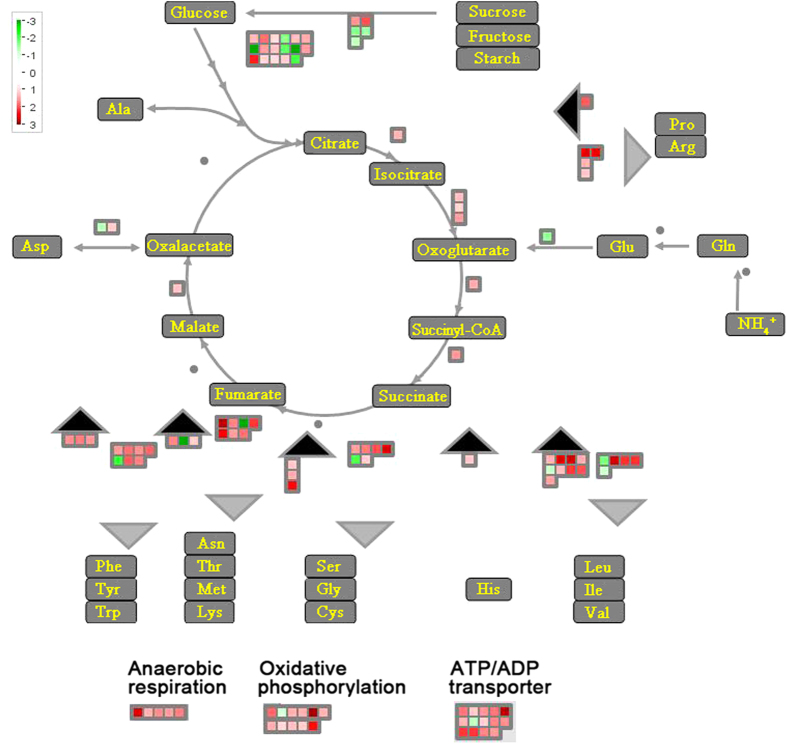
DEGs in litchi fruit after pre-cold storage. After cluster analysis, genes related to energy metabolism were selected, and genes that were specifically expressed in PCS fruits were graphically analysed and visualised using the MapMan database at TAIR (http://www.arabidopsis.org/). All genes are shown in small squares. Up-regulated genes are presented in red and down-regulated genes in green.

Energy production and conversion genes related to NADPH synthesis (lychee_10129), ATP transport (lychee_14863) and adenosine 5′-triphosphatase (ATPase) activity (lychee_34156) were up-regulated during litchi fruit senescence. Specifically, 78 energy production and conversion genes were up-regulated in the PCS fruit (Fig. 7). Among these genes involved in ATP synthesis and nicotinamide-adenine dinucleotide (NADH) synthesis and reduction, a large number of their corresponding proteins are predicted to participate in energy production and conversion (Fig. 7). Interestingly, three ATP transport-related genes, which were predicted to be members of the ABC transporter family, were up-regulated in the PCS fruit: lychee_5370, lychee_49172 and lychee_52807 (Fig. 7). The expression profiles of these genes suggest that more energy was consumed during PCS fruit senescence than AC fruit senescence.

The tricarboxylic acid cycle is a series of enzyme-catalysed chemical reactions that form a key pathway in aerobic plant respiration. Genes predicted to be malate dehydrogenases were up-regulated in both AC and PCS fruits. Specifically, ten malate dehydrogenase genes (six isoforms), isocitrate dehydrogenase (three isoforms) and 2-oxoglutarate dehydrogenase (one isoform) were up-regulated, but only in the PCS fruit (Fig. 7). In addition, the aldehyde dehydrogenase gene, which encodes a critical enzyme for anaerobic respiration, was up-regulated; one isoform was up-regulated in the AC fruit and five isoforms in the PCS fruit (Fig. 7). These findings suggest that both aerobic and anaerobic respiration processes were activated in senescent AC and PCS fruits but were more pronounced in the PCS fruit.

### Intracellular energy metabolism molecules that were differentially accumulated during litchi fruit senescence

**Figure 8:**
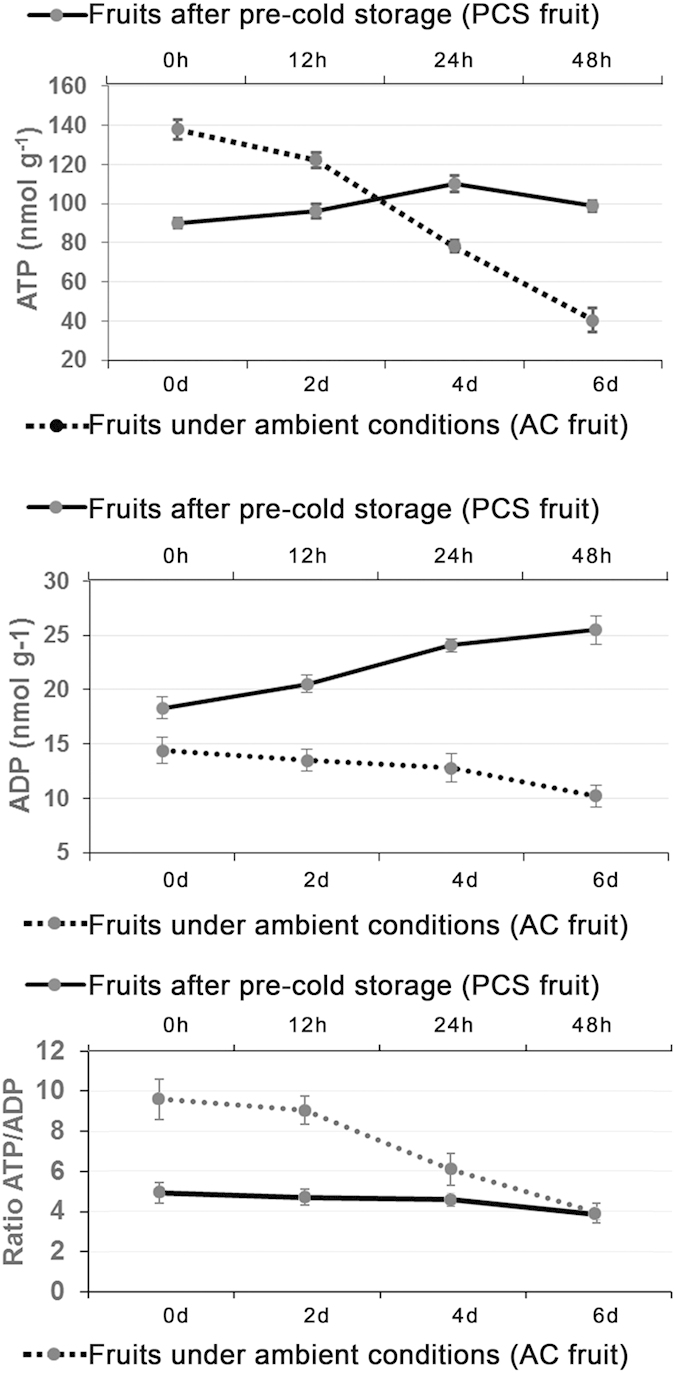
Energy status in senescent litchi fruits during ambient storage and shelf life after pre-cold storage. ATP and ADP contents are expressed as nmol g^−1^ FW (fresh weight). The energy statuses of PCS and ACS fruits are shown as solid and dotted lines, respectively. AC fruits were stored under ambient conditions (approximately 20–25 °C and 75–85% relative humidity) immediately after harvest. After cold storage (4 °C and 75–85% relative humidity) for 14 days, PCS fruits were also stored under ambient conditions at 20–25 °C and 75–85% relative humidity. The standard errors are presented using error bars.

To examine the roles of molecules involved in intracellular energy metabolism during litchi fruit senescence, ATP and ADP contents were analysed. ATP and ADP levels decreased during AC fruit senescence but increased in senescent PCS fruits (Fig. 8). Interestingly, the ATP/ADP ratio decreased gradually during the senescence of AC and PCS fruits; however, the ratio was lower in the PCS fruit than in the AC fruit, especially at the time of harvest of the AC fruits, compared with the PCS fruits at 0 h after 14 days of cold storage (Fig. 8).

### Verification by real-time qRT-PCR

**Figure 9:**
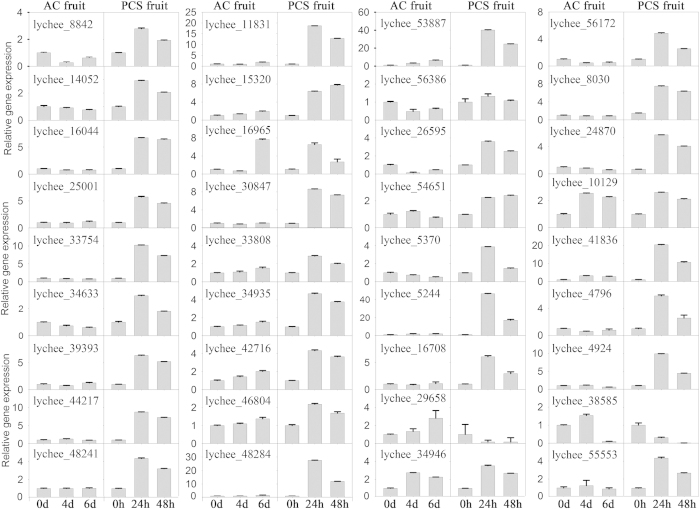
Verification of gene expression. The transcript levels of 36 selected genes were determined at different stages of litchi fruit senescence. The relative expression levels were analysed by quantitative real-time PCR. The default gene expression level was set to 1 at 0 days in AC fruit and 0 h in PCS fruit. The data for each gene are presented on a single graph and the y-axis is shared by the results for the AC and PCS fruit.

To verify the usability of the transcriptomic data, the relative transcript levels of 36 genes that were either significantly up- or down-regulated were determined using quantitative real-time reverse-transcription PCR (qRT-PCR) (Fig. 9). These genes included nine ABA signal-related genes (lychee_15320, lychee_16044, lychee_42716, lychee_25001, lychee_16965, lychee_34633, lychee_56386, lychee_56172 and lychee_8842), three calcium-mediated signalling-related genes (lychee_44217, lychee_53887 and lychee_33754), three *G* protein-coupled receptor signalling-related genes (lychee_11831, lychee_26595 and lychee_34935), nine GTPase signal-related genes (lychee_8030, lychee_14052, lychee_24870, lychee_30847, lychee_33808, lychee_39393, lychee_46804, lychee_48241 and lychee_48284), chloroplastic linoleate 13S-lipoxygenase 3-1, (lychee_54651), malate dehydrogenase (lychee_10129), a predicted ABC transporter C family member 2 isoform 1 (lychee_5370), a predicted protein (lychee_41836), phenylalanine ammonia lyase (lychee_5244), a putative cytochrome P450 (lychee_47963), long-chain acyl-CoA synthetase 4 (lychee_16708), sphingolipid fatty acid alpha hydroxylase (lychee_4924), an acidic endochitinase-like protein (lychee_29658), a putative ATP-binding protein (lychee_38585), a serine/threonine protein kinase (lychee_55553), a putative class I chitinase (lychee_51483) and xyloglucan endotransglycosylase (lychee_34946). These genes are involved in signal transduction, transport, cell wall function, respiration and energy, and fatty acid and other types of metabolism. The results obtained by qRT-PCR correlated well with the transcriptomic data, suggesting that the transcriptomic data were accurate and useful.

## Discussion

### Litchi fruit senescence

Litchi is a non-climacteric subtropical fruit that is highly valued commercially for its white, translucent aril and attractive red skin colour. However, harvested litchi fruit deteriorate quickly due to rapid pericarp browning, leading to a reduced market value^15^. Cold storage effectively slows down litchi pericarp browning and reduces TSS loss. However, it has been noted that the subsequent shelf life of the fruit after cold storage is <48 h. Pericarp browning occurs faster in PCS fruit than in AC fruit under ambient temperature conditions, which suggests that there are factors that might induce and accelerate the browning. Fruit senescence is the final event in fruit development and is regulated by internal and external factors. The senescence of non-climacteric fruit is a complicated process that involves an entirely controlled programme that is coordinated by internal signals to achieve maximum efficiency for life support^9^.

### Transcriptome of litchi pericarp

The transcriptome of the developing litchi pericarp was assembled by Li *et al.* in 2014 using the cultivar “Baitangying”, with special attention paid to pericarp cracking^16^. In the present study, the transcriptome of the senescent pericarp using the “Huaizhi” cultivar was assembled, with special attention paid to pericarp browning, suggesting that the two transcriptome libraries are different. Therefore, it has been kindly suggested that this study is the first report of comparative transcriptome profiling of litchi fruit browning.

### *G* protein-coupled receptor protein-, small GTPase- and calcium ion signal- accelerated litchi fruit senescence

Plant hormones have long been implicated in the regulation of fruit senescence. ABA may play a key role in initiating non-climacteric fruit senescence^8^. In the present study, the senescence of both the AC and PCS fruits was regulated by an ABA-mediated signalling pathway. However, more genes involved in senescence-related responses to ABA stimulation and the ABA-mediated signalling pathway were up-regulated in the PCS fruit than in the AC fruit (Fig. 6).

Additionally, calcium ions play important roles in the induction of cell death and as secondary messengers during cold-induced signal transduction via a stimulus-specific increase in [Ca^2+^]_cyt_^17^. The calcium-sensing receptor is a class C G protein-coupled receptor that senses the level of calcium ions^18^, whereas the small GTPase positively regulates calcium ion signalling^19^. During PCS fruit senescence, many of these genes were up-regulated, including seven genes involved in calcium-mediated signalling, four genes that operate in the G protein-coupled receptor signalling pathway, and 20 genes involved in small GTPase-mediated signal transduction (Fig. 6). Interestingly, during the accelerated PCS fruit senescence, genes involved in calcium ion-, G protein-coupled receptor- and GTPase-mediated signalling were dramatically up-regulated. Thus, the results suggested that calcium ion-, G protein- and GTPase-mediated signal transduction might be involved in the acceleration of PCS fruit senescence.

### Oxidation-related genes might play roles during litchi fruit senescence

ROS play a critical role in fruit senescence^11^, and cold stress can increase ROS production^20^. This study found that cold storage of litchi fruit induced the expression of respiratory burst oxidases that encode the key subunits of plant NADPH oxidases, which catalyse the reduction of molecular oxygen to produce hydrogen peroxide^21^. NADPH oxidase activity can be controlled by small GTPases of the Rop family^22^. These findings suggest that pre-cold storage increased ROS production via a small GTPase- signalling pathway in the PCS fruit. Enhanced ROS production can pose a threat to cells by causing lipid peroxidation, protein oxidation, nucleic acid damage and activation of the programmed cell death pathway, which ultimately lead to cell death^10^. ROS production could be one of the causes of accelerated senescence in the pre-cold storage fruit.

Lipid peroxidation is an inherent feature of senescing cells^12^ and can be triggered by ROS or lipoxygenase during plant cell senescence. In senescing AC fruit, lipoxygenase was down-regulated; therefore, lipid peroxidation is mediated by ROS alone. In contrast, PCS fruit senescence was promoted by ROS and lipoxygenase, which is consistent with accelerated senescence initiated by pre-cold storage.

Pericarp browning is an important characteristic of fruit senescence. It has been well established that litchi pericarp browning is generally attributable to the oxidation of phenolic compounds^14^,^23^. Elevated levels of phenolics in the cells cause slowing of senescence^14^. However, more putative peroxidase genes were up-regulated during PCS fruit senescence than during AC fruit senescence. Phenolic compounds can be oxidised to corresponding semiquinones and quinones by peroxidases^24^. In addition, anthocyanin oxidation requires that these molecules become accessible to peroxidases. The major litchi anthocyanin cyanidin 3-rutinoside can be oxidised by litchi peroxidases^25^.

In short, litchi senescence might be accompanied by the oxidation of fatty acids, polyphenols and anthocyanins, processes that are mediated by ROS and peroxidases. Pre-cold storage not only increased ROS production but also up-regulated peroxidases that could collectively oxidise fatty acids, anthocyanins and phenolics in senescing litchi fruit. The additional expression of genes associated with the lipoxygenase oxidation pathway in the PCS fruit might further accelerate the senescence process.

### Genes related to phenylalanine metabolism might play vital roles during litchi fruit senescence

Phenylpropanoid metabolic processes represent the principal pathways of anthocyanin and flavonoid synthesis. The major polyphenols present in litchi fruit were flavonoids^26^. A large number of genes in the phenylpropanoid metabolic pathway linked with cytochrome P450 were up-regulated during fruit senescence but more so in the PCS fruit than in the AC fruit (Supplementary Excel S1), which might lead to enhanced polyphenol synthesis. The increased polyphenol levels could then be oxidised by ROS and peroxidases to cause pericarp browning. Pre-cold storage may have induced the accumulation of more polyphenols in the PCS fruit than in the AC fruit during storage under ambient temperature conditions, and the subsequent oxidation may have caused the accelerated fruit senescence. Plant cytochrome P450s are involved in a wide range of biosynthetic reactions that lead to the production of various fatty acid conjugates, plant hormones, defensive compounds or medically important drugs^27^. Up-regulated cytochrome P450s could promote litchi fruit senescence through signal regulation and primary and secondary metabolism. However, which process plays the more important role needs to be examined in a future study. In the present study, polyphenol oxidase, an important enzyme in litchi pericarp browning^26^, was not detected in either AC or PCS fruit, possibly because the sequencing depth was less.

In higher plants, coumarins originate from the general phenylpropanoid pathway, and they can inhibit the growth of rice, mung bean, lettuce and clover seedlings^28^. Coumarins also inhibit ^14^C-glucose incorporation into cellulose by inhibiting its biosynthesis rather than its breakdown^28^. In addition, coumarins abolish the exponential phase and accelerate the onset of the stationary phase of cell growth. These allelochemical compounds may also act as inhibitors of the cell cycle and/or as senescence-promoting substances^29^. Most of the coumarin-related synthesis genes were up-regulated in both the AC and PCS fruits, but more so in the PCS fruit. On these grounds, it is speculated that coumarins might play a role in promoting fruit senescence, but this idea needs to be confirmed.

### Protein phosphorylation might play a vital role during litchi fruit senescence

Protein activities can be regulated in many ways. Protein phosphorylation has emerged as a major regulatory mechanism in plant signalling and senescence^30^. In the present study, serine/threonine protein kinases and calcium-dependent protein kinases were up-regulated in the PCS fruit specifically, possibly contributing to their accelerated senescence. Phosphorylation and dephosphorylation cascades can be triggered by calcium ion signalling^31^. Consistent with our results, calcium-dependent protein kinases also regulate ROS production by NADPH oxidase^32^. Thus, calcium could function as both a signalling molecule and an inducer of the oxidative burst.

### Genes and proteins related to energy metabolism play vital roles during litchi fruit senescence

In the cell, the rate of mitochondrial oxidative phosphorylation is tightly regulated by the ATP/ADP ratio^33^, such that low ATP/ADP ratios reflect cellular energy deficiencies rather than low ATP concentrations. Thus, the PCS fruit at 0 h in the ambient temperature after 14 days of cold storage exhibited greater energy deficiency, as indicated by the significantly lower ATP/ADP ratio than the ratio found in the AC fruits at 0 days (Fig. 8). To compensate for this energy deficiency, genes involved in the tricarboxylic acid cycle were greatly up-regulated, including oxidative phosphorylation, anaerobic respiration, ATP synthesis and ABC transporter genes (Supplementary Excel S1). Furthermore, increased monosaccharide levels in the PCS fruit compared with the AC fruit provided more substrates for the tricarboxylic acid cycle (Fig. 5). These findings were consistent with gradual AC fruit senescence and accelerated PCS fruit senescence that was driven by an energy deficiency^34^.

## Conclusion

**Figure 10:**
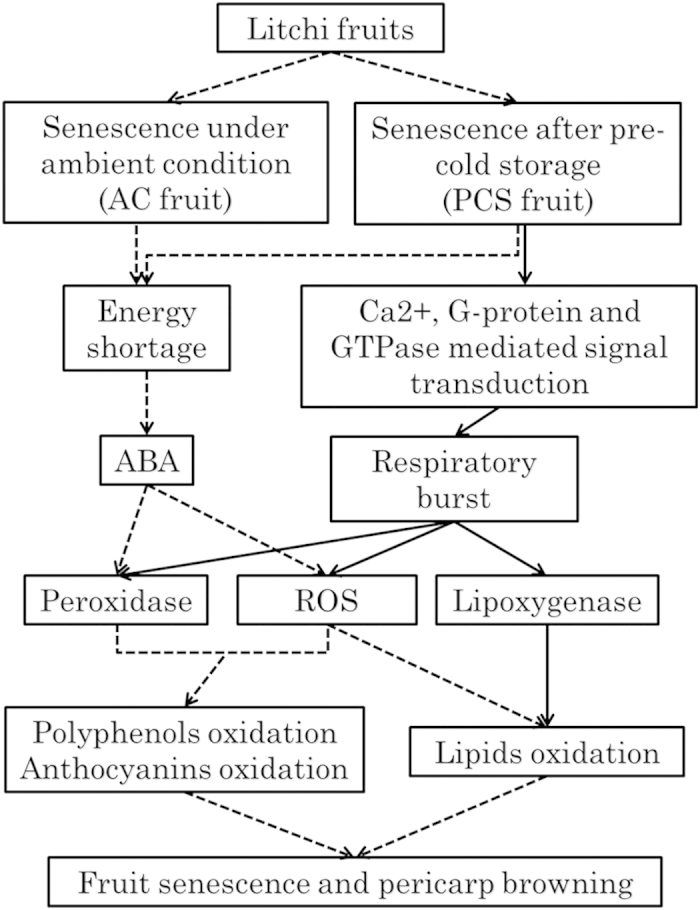
A speculative model of litchi fruit senescence. Litchi fruit senescence is likely to be an oxidation process regulated by ABA under ambient conditions. After pre-cold storage, the senescence process was accelerated. This model is based on the reported transcriptome and metabolome findings. Solid arrows indicate activation that is specific to PCS fruits. Arrows with broken shafts indicate events that occur in both AC and PCS fruits.

This study provided an overall picture of litchi fruit senescence. The browning index, TSS and respiration rate showed that cold storage effectively slows the pericarp browning of litchi and reduces TSS loss and suggests that the critical turning points in fruit senescence were 4 days for the AC fruit and 24 h for the PCS fruit. Comparative transcriptomic and metabolomic analyses provided new insights into how litchi fruit senescence was accelerated by pre-cold storage. Under ambient conditions, litchi fruit senescence was likely to be an oxidation process promoted by ABA, including the oxidation of lipids, polyphenols and anthocyanins driven by up-regulated peroxidase activity and increased ROS production (Fig. 10). The accelerated senescence in PCS fruit could might be due to up-regulated calcium signal-, *G* protein-coupled receptor- and small GTPase-mediated signal transduction, which elicited a respiratory burst. The respiratory burst could have led to increased ROS production, up-regulated peroxidase activity and the initiation of the lipoxygenase pathway, which would, in turn, drive the accelerated senescence of the PCS fruit (Fig. 10).

## Materials and Methods

### Sample collection

Mature litchi (*Litchi chinensis* Sonn., cv. Huaizhi) fruits were obtained from a commercial orchard in Guangzhou, China. Fruits were selected for uniformity in shape, size and colour and were divided into two groups. One group was stored under ambient temperature conditions (AC) (20−25 °C and 75−85% relative humidity) and was sampled at 0, 2, 4, 6 and 8 days. The other group of fruit was stored at a cold temperature (PCS) (4 °C and 75−85% relative humidity) for 14 days. After cold storage, the PCS fruit was stored under the same ambient temperature conditions as the AC fruit and was sampled at 0, 12, 24, 36 and 48 hours. The pericarp tissue from each fruit was surgically removed along the equatorial plane; 30 fruits formed one biological replicate, and five biological replicates were performed for each sampling time. Each pericarp replicate was ground to powder in liquid nitrogen and then stored at −80 °C until needed for further analyses.

### Determination of the characteristics of litchi fruit senescence

Important indices of senescence in litchi fruit, including the browning index, respiration rate and total soluble solids (TSS), were determined in the present study. The browning index was assessed by evaluating the browned area on each pericarp of more than 120 fruits, according to Duan *et al.*^23^. The following browning scale was used: 0 = no browning (excellent quality); 1 = slight browning; 2 = <1/4 browning; 3 = 1/4−1/2 browning; and 4 = >1/2 browning. The browning index was calculated with this equation: ∑ (browning scale × number of corresponding fruits)/4 × total number of fruits. One replicate composed of 30 litchi fruits was used for measurements of the respiration rate by infrared gas analysis using an LI-6262 CO_2_/H_2_O analyser (LI-COR, Inc., Lincoln, NE, USA) according to the method of Wang *et al.*^4^. Juice was obtained from the fruit flesh with a juicer (HR1861, Philips Co., Beijing, China) and was then filtered through cheesecloth. The TSS content of the juice was determined using a portable refractometer (Atago PAL-1, Japan).

### RNA extraction, library preparation and sequencing

Total RNA was extracted from pericarp tissues using a Qiagen RNeasy Kit according to the manufacturer’s instructions. Zero and 4 day samples of AC fruit and 0, 24 and 48 h samples of PCS fruit were used for RNA library preparation and sequencing. RNA degradation and contamination was monitored on 1% agarose gels. RNA purity was checked using a NanoPhotometer^®^ spectrophotometer (Implen, CA, USA). The RNA concentration was measured using the Qubit^®^ RNA Assay Kit with a Qubit^®^ 2.0 Fluorometer (Life Technologies, CA, USA). RNA integrity was assessed using the RNA 6000 Nano Assay Kit with the Bioanalyzer 2100 system (Agilent Technologies, CA, USA). Messenger RNA (mRNA) was purified from total RNA using polyT oligos attached to magnetic beads. Fragmentation was achieved using divalent cations under elevated temperatures in a proprietary fragmentation buffer (Illumina). The first-strand cDNA synthesis was performed using random oligonucleotides and SuperScript II. The second-strand cDNA synthesis was performed using DNA polymerase I and ribonuclease H. After adenylation of the 3′-ends of the DNA fragments, Illumina PE adapter oligonucleotides were ligated to prepare for hybridisation. To preferentially select cDNA fragments of 200 base pairs (bp) in length, the library fragments were purified with the AMPure XP system (Beckman Coulter, Beverly, USA). DNA fragments with ligated adaptor molecules on both ends were selectively enriched using the Illumina PCR Primer Cocktail in a 10-cycle PCR. Products were purified using the AMPure XP system and were quantified using the Agilent High Sensitivity DNA assay with the Agilent Bioanalyzer 2100 system. The clustering of the index-coded samples was performed on a cBot System using the TruSeq PE Cluster Kit v3-cBot-HS (Illumina) according to the manufacturer’s instructions. After cluster generation, the library preparations were sequenced on an Illumina HiSeq 2000 platform. Two biological samples from each time point were used for library construction. One sample was paired-end sequenced (expected library size: 200 bp; read length: 100 nucleotides (nt)); the other was single-read sequenced (expected library size: 200 bp; read length: 40 nt). Each library was sequenced once. The raw data were uploaded to the National Center for Biotechnology Information (NCBI, SRA247016).

### *De novo* sequence assembly and functional annotation of the transcriptome

Raw data (raw reads) in FASTQ format were first processed through Perl scripts that we developed. In this step, clean data (clean reads) were obtained by removing reads containing adaptors and poly-N and low-quality reads from the raw data. At the same time, the Q20, GC content and sequence duplication level of the clean data were calculated. All the downstream analyses were conducted on high-quality, clean data. Paired-end sequencing was performed. The left files (read 1 file) from all libraries/samples were pooled into one large left.fq file, and the right files (read 2 file) were pooled into one large right.fq file. Transcriptome assembly was performed based on the left.fq and right.fq files using Trinity with min_kmer_cov set to 2 and all other parameters set to default^35^. In the last step, blastx (E-value < 0.00001) was employed to search for homologues of our assembled unigenes in protein databases such as NR, Swiss-Prot, KEGG and COG. The best results were used to determine the sequence orientations of the unigenes. If results from different databases conflicted with one another, a priority order (i.e., NR, Swiss-Prot, KEGG and COG) was followed to determine the sequence orientation. If a unigene aligned to none of the above databases, ESTScan software was used to predict its coding regions and to determine their sequence orientations^36^. The functional annotation by GO terms (http://www.geneontology.org) was analysed using the program Blast2GO. The COG and KEGG pathway annotations were performed using Blastall software against the COG and KEGG databases, respectively.

### Differential unigene expression

The uniquely mapped reads for a specific transcript were counted by mapping them to the assembled sequences using SOAP^37^. The gene expression level was calculated using RPKM^38^. The RPKM value for each transcript was then measured in reads per kilobase of transcript sequence per million mapped reads^38^. Furthermore, the fold changes in transcript levels were calculated using this formula: log2 (case_RPKM/control_RPKM). If the value of either the case_RPKM or the control_RPKM was zero, 0.01 instead of zero was used to calculate the fold change. The false discovery rate method was used to determine the threshold of the *P* value in multiple tests. ‘FDR ≤ 0.001 and an absolute value of log2Ratio ≥ 1’ was the threshold used to judge the significance of the observed differential gene expression. Paired-end and single-read sequenced data were used to determine which genes were differentially expressed, respectively. Genes that exhibited significant differential expression (between two samples) in both of the sequencing approaches were used for further analysis.

### GO functional enrichment analysis for differentially expressed genes (DEGs)

The analysis mapped all DEGs to GO terms in the database by calculating the gene numbers for every term, followed by an ultra-geometric test to find significantly enriched GO terms among the DEGs compared with the transcriptome background. GO terms that fulfilled this condition were then defined as significantly enriched GO terms among the DEGs. Finally, the Blastall program was used to annotate the pathways represented by the DEGs using the KEGG database.

### Real-time quantitative RT-PCR verification

Zero-, 4- and 6-day samples of AC fruit and 0-, 24- and 48-h samples of PCS fruit were used for real-time quantitative RT-PCR verification. Two micrograms of total RNA was reverse-transcribed to obtain first strand cDNA using the RevertAid First Strand cDNA Synthesis Kit (Fermentas, Lithuania) according to the manufacturer’s instructions. Gene-specific primer pairs had been designed using Primer Express software (Applied Biosystems, Foster City, CA, USA) were used for real-time PCR that was run on the ABI 7500 Real-Time System (PE Applied Biosystems, Foster City, CA, USA). Actin was used as the standard to normalise the cDNA content^4^.

### Primary metabolic profiling

PCS fruit samples (0, 24 and 48 h) and AC fruit samples (0, 4 and 6 days) were used for primary metabolic profiling analysis. Metabolites were detected using gas chromatography coupled with mass spectrometry (GC-MS) according to the method of Yun *et al.*^39^. A total of 300 mg of each fruit sample was extracted in 2700 μl of methanol, and 300 μl of 0.2 mg ml^−1^ ribitol in water was added as an internal standard. The following MS operating parameters were used: an ionisation voltage of 70 eV (electron impact ionisation); an ion source temperature of 200 °C; and an interface temperature of 250 °C. Spectra of the total ion current were recorded in the mass range of 45−600 atomic mass units in the scanning mode.

### Determination of ATP and ADP concentrations

Measurements of the ATP and ADP contents were performed according to the method of Wang *et al.* using a Waters 2695 HPLC (Waters, Inc., Milford, MA, USA) with a Pinnacle II C18 column (4.6 × 250 mm) and an ultraviolet detector set to 254 nm^4^.

### Statistical analyses

Five biological replicates were used to measure the pericarp browning index, respiration rate, TSS and ATP and ADP contents, and four biological replicates were used for GC-MS and qRT-PCR analysis. Data for each sample were statistically analysed using Student’s *t*-test (*P* < 0.05).

## Additional Information

**How to cite this article**: Yun, Z. *et al.* Comparative transcriptome and metabolome provides new insights into the regulatory mechanisms of accelerated senescence in litchi fruit after cold storage. *Sci. Rep.* **6**, 19356; doi: 10.1038/srep19356 (2016).

## Supplementary Material

Supplementary Information

Supplementary Excel table S1
