# Supplementary material for: Comparative transcriptome and metabolome provides new insights into the regulatory mechanisms of accelerated senescence in litchi fruit after cold storage
Source: Sci Rep. 2016 Jan 14;6:19356. doi: 10.1038/srep19356 (PMC4725888; doi:10.1038/srep19356)
Supplement: Supplementary Information [file srep19356-s1.pdf]

Comparison of the litchi transcriptome and metabolome provides new insights into the regulatory mechanisms of accelerated senescence in the fruit after cold storage

Running title: Accelerated litchi fruit senescence after pre-cold storage.

Ze Yun,[†1] Hongxia Qu,[†1] Hui Wang,[1] Feng Zhu,[2] Zhengke Zhang,[1] Xuewu Duan,[1] Bao Yang,[1] Yunjiang Cheng[2] and Yueming Jiang*[1]

[1] Key Laboratory of Plant Resources Conservation and Sustainable Utilization, South China Botanical Garden, Chinese Academy of Sciences, Guangzhou 510650, P.R. China

[2] Key Laboratory of Horticultural Plant Biology, Ministry of Education, Huazhong Agricultural University, Wuhan 430070, P.R. China

*Corresponding author: Yueming Jiang

Email address: ymjiang@scbg.ac.cn

Phone number: +86-2037083042;

Fax number: +86-2037252525

Author Contributions:

[†] Ze Yun and Hongxia Qu contributed equally to this work.

Supplementary information

Supplementary Table S1. Summary of the statistics for sequence data from the litchi fruit pericarp under different storage conditions. AC fruit after 0 and 4 days of cold storage and PCS fruit after 0, 24 and 48 h at ambient temperature were used to construct two libraries. One library was paired-end sequenced (expected library size of 200 bp and read length of 100 nucleotides), and the other was single-read sequenced (expected library size of 200 bp and read length of 40 nt). Each library was sequenced once.

**Summary**		**AC 0 d**		**AC 4 d**		**PCS 0 h**		**PCS 24 h**		**PCS 48 h**	
		**single**	**paired**	**single**	**paired**	**single**	**paired**	**single**	**paired**	**single**	**paired**
Raw Data	Total	42393892	8235846	35730419	6633639	44270771	7306256	38147766	7752975	44554246	8149186
Clean Reads	Total number	39002380	6671035	33943898	5837602	40286401	6356442	36240377	6434969	40989906	6682332
All Reads Mapping to Gene	Total number	37432913	5073839	31625604	4688489	38741516	5022716	33559720	5397477	39272065	5742475
All Reads Mapping to Gene	Total % of clean tag	95.98	76.06	93.17	80.32	96.17	79.02	92.60	83.878	95.81	85.94

Supplementary Figure S1. Litchi pericarp browning in the AC and PCS fruits.

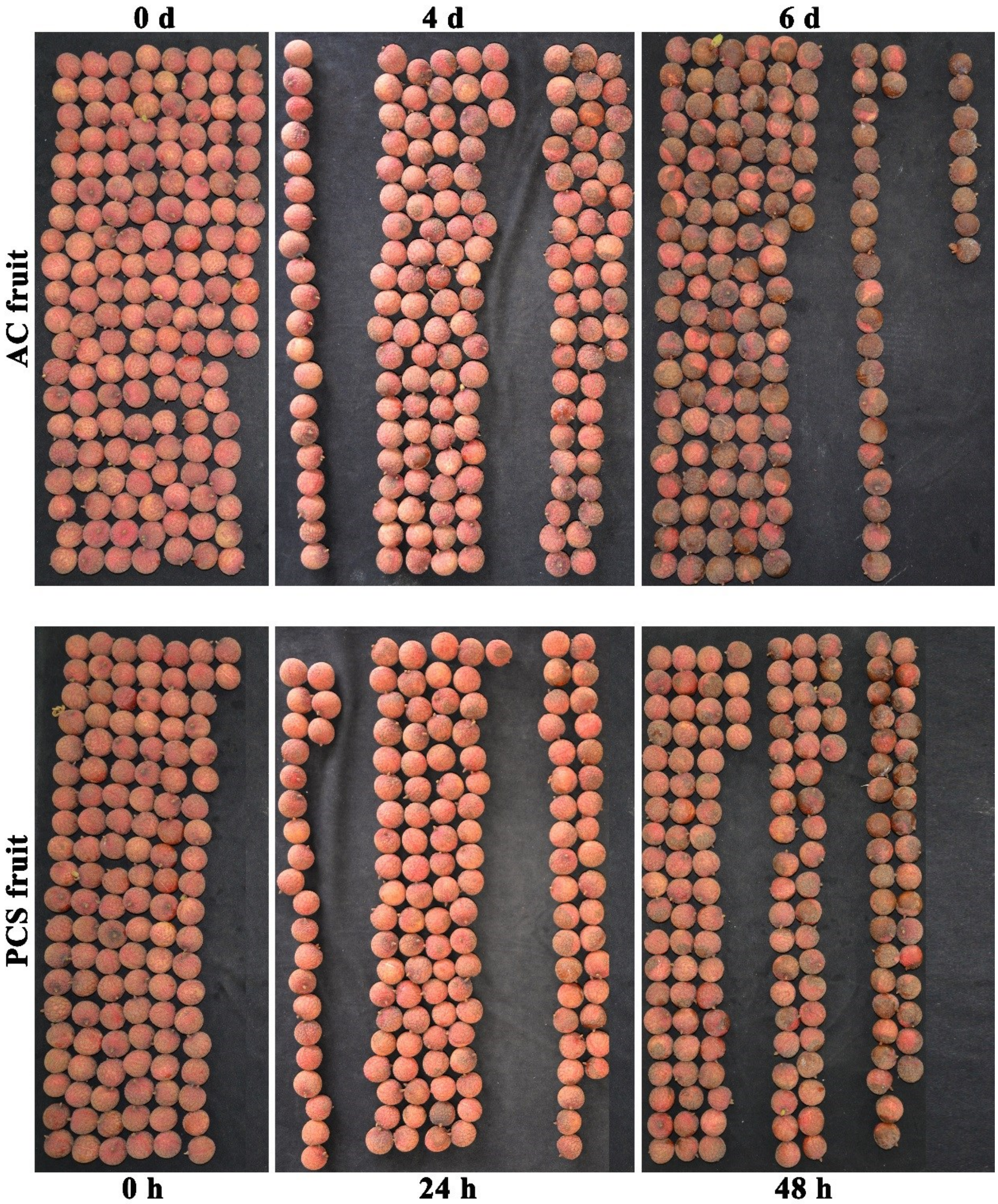

Supplementary Figure S2. Graphical output of up-regulated genes related to signal transduction during PCS fruit senescence. GO annotations were performed on DEGs identified in the transcriptome. Up-regulated genes related to signal transduction in the PCS fruit were analysed and visualised as a directed acyclic graph using the BiNGO plugin in Cytoscape. Categories are shown as circles, and the diameters represent the number of genes in each category. Areas coloured deep yellow represent significant nodes, whereas the white areas represent the nodes that are not significant.

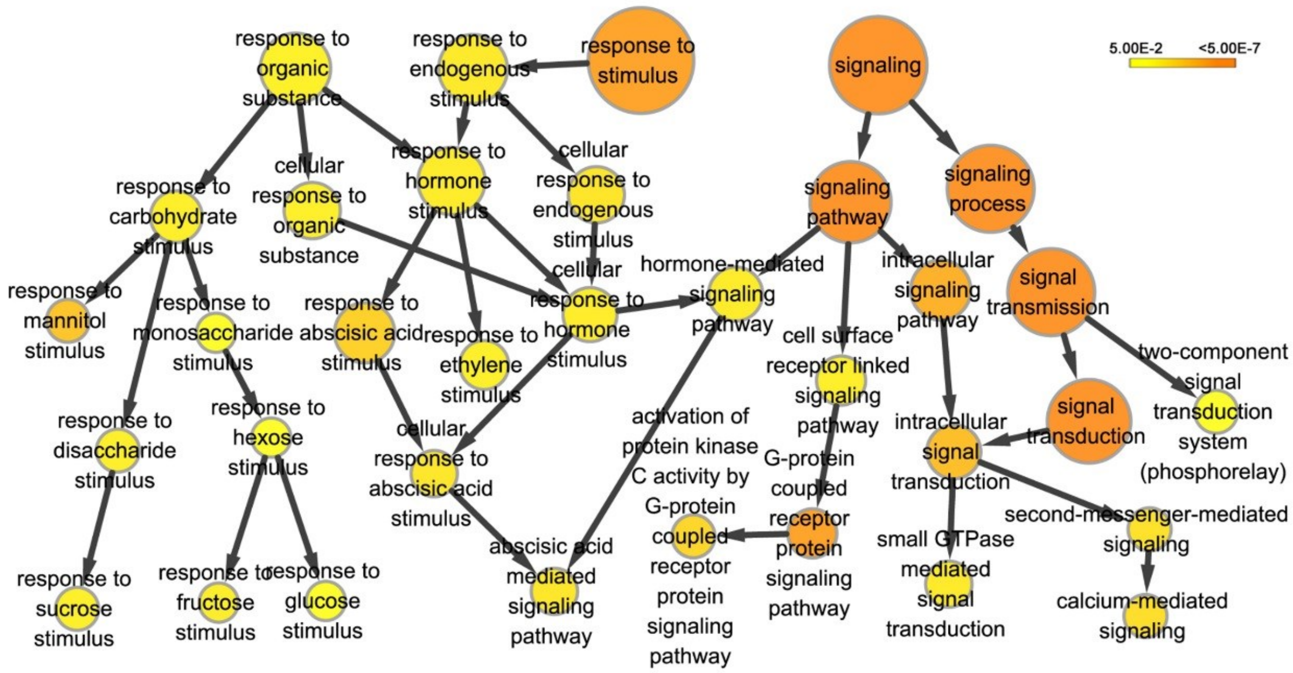

Supplementary Figure S3. Graphical output of up-regulated genes related to secondary metabolism in senescent AC and PCS fruits. GO annotations were performed on DEGs identified in the transcriptome. Up-regulated genes related to secondary metabolism were analysed and visualised in a directed acyclic graph using the BiNGO plugin in Cytoscape. Categories are shown as circles, and the diameters represent the number of genes in the category. Deep yellow areas represent significant nodes, whereas white areas represent nodes that are not significant.

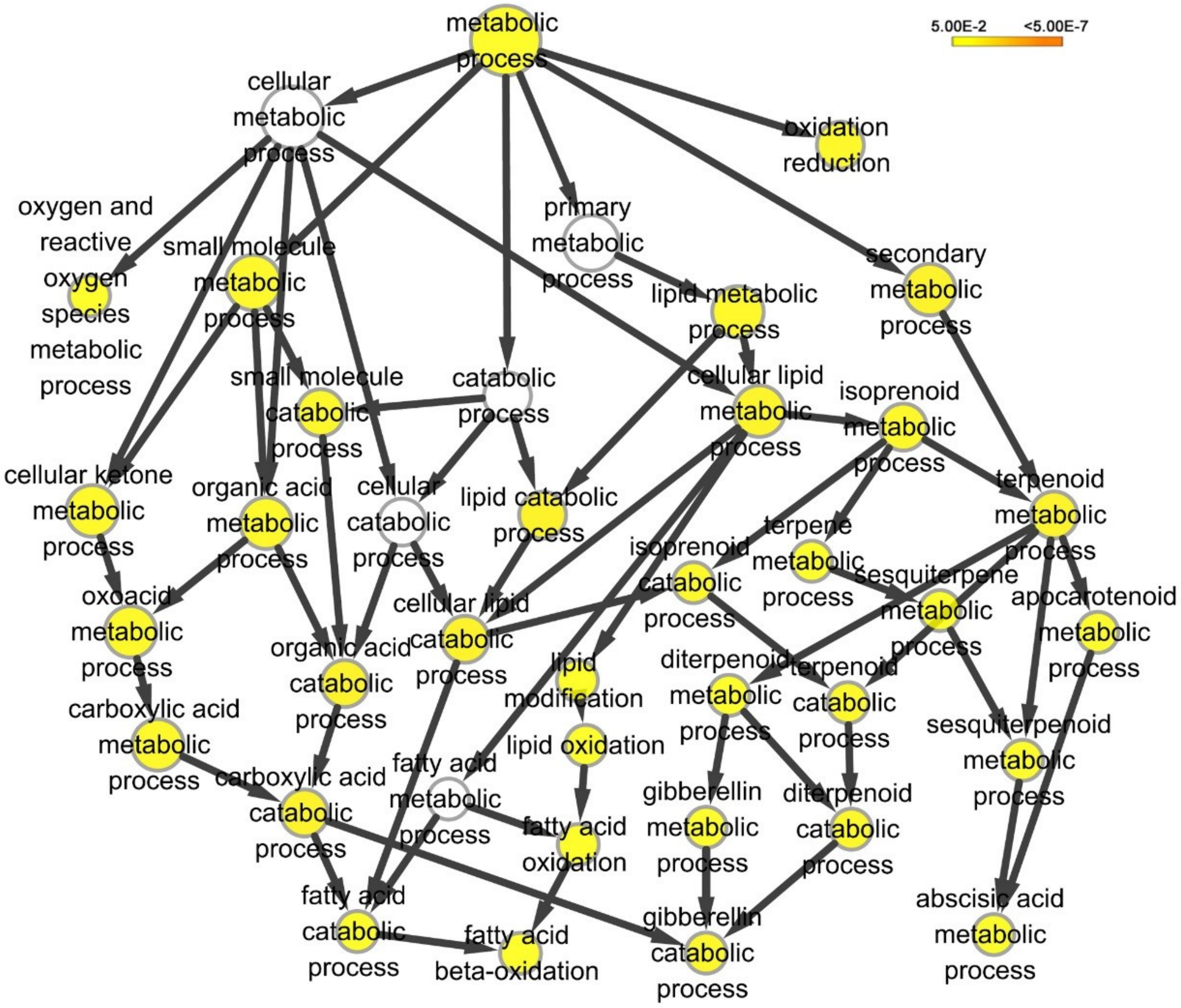

Supplementary Figure S4. Graphical output of up-regulated genes related to lipid metabolism during senescence of the PCS fruit. GO annotations were performed on DEGs identified in the transcriptome. Up-regulated genes related to lipid metabolism in the PCS fruit were analysed and visualised in a directed acyclic graph using the BiNGO plugin in Cytoscape. Categories are shown as circles, and the diameters represent the number of genes in each category. Deep yellow areas represent significant nodes, whereas white areas represent nodes that are not significant.

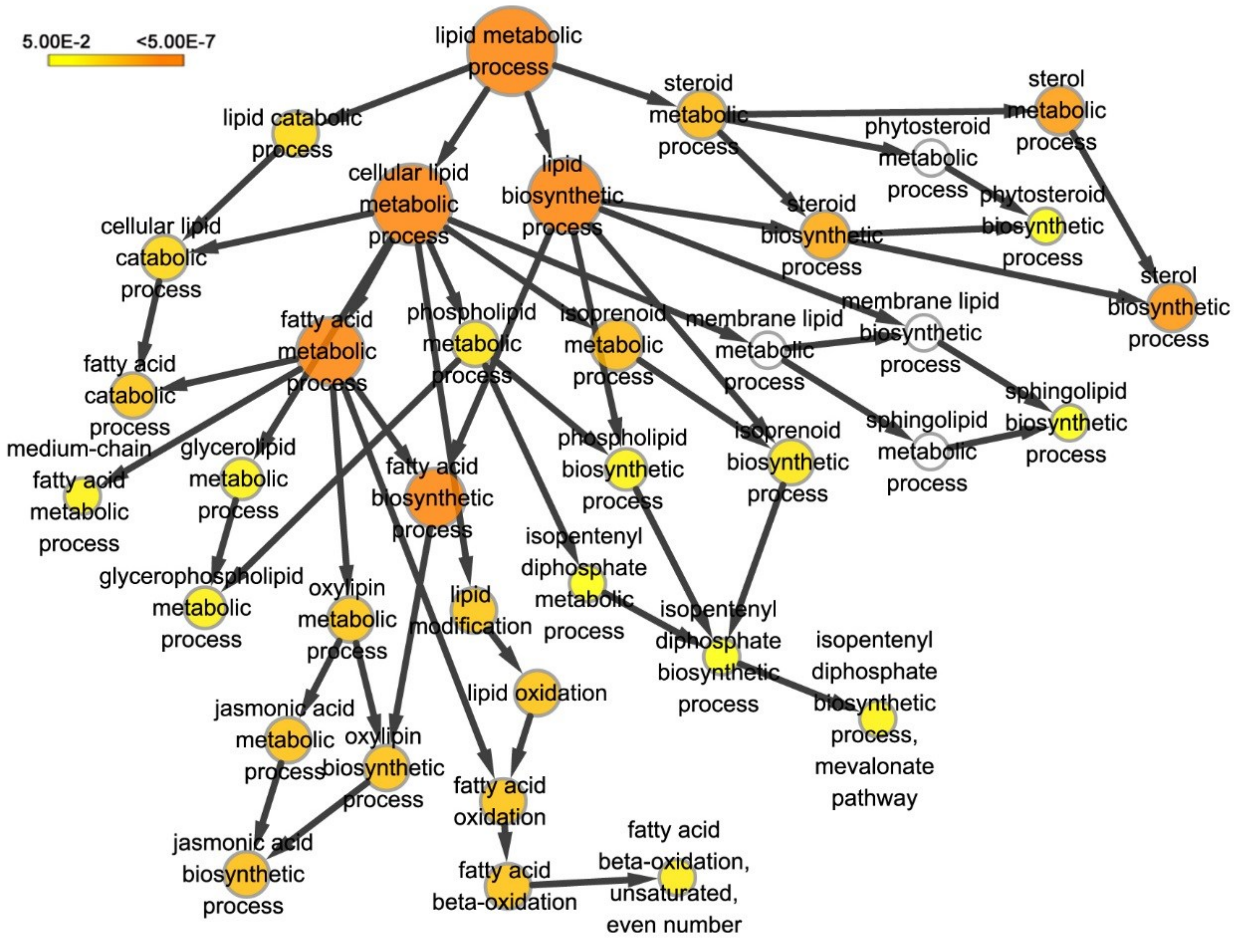

Supplementary Excel table S1. DEGs during the senescence of litchi fruit. AC fruits were stored under ambient temperature conditions (approximately 20-25 °C and 75-85% relative humidity) immediately after harvest. After 14 days of cold storage (4 °C and 75-85% relative humidity), PCS fruits were stored under the same ambient temperature conditions mentioned above.
